# An Eco-Friendly Extraction and Purification Approach for Obtaining Active Ingredients for Cosmetics from Two Marine Brown Seaweeds

**DOI:** 10.3390/md22030112

**Published:** 2024-02-28

**Authors:** Leslie Gager, Solène Connan, Stéphane Cérantola, Sylvain Petek, Céline Couteau, Laurence Coiffard, Valérie Stiger-Pouvreau

**Affiliations:** 1 Univ Brest, CNRS, IRD, Ifremer, LEMAR, IUEM, F-29280 Plouzane, France; leslie.gager@gmail.com (L.G.); solene.connan@univ-brest.fr (S.C.); sylvain.petek@ird.fr (S.P.); 2 Univ Brest, Plateforme RMN-RPE, F-29200 Brest, France; stephane.cerantola@univ-brest.fr; 3 Faculté de Pharmacie, Université de Nantes, LIEN, EA4685, F-44000 Nantes, France; celine.couteau@univ-nantes.fr (C.C.); laurence.coiffard@univ-nantes.fr (L.C.)

**Keywords:** active and valuable ingredient, cosmetic, green extraction and purification, macroalgae, NMR follow-up, polyphenols

## Abstract

Brown seaweeds are attracting attention due to their richness in bioactive compounds, in particular, their phlorotannins. We present here a case study of two Fucales, *Ascophyllum nodosum* and *Halidrys siliquosa*, sustainably collected, to produce active polyphenols for the cosmetics sector. Phenolic contents of crude extracts, obtained by Accelerated Solvent Extraction (ASE), were more elevated in *H. siliquosa* at 100.05 mg/g dry weight (DW) than in *A. nodosum* (29.51 mg/g DW), considering 3 cycles with cell inversion. The temperature of extraction for a high phenolic content and high associated antioxidant activities close to positive controls was 150 °C for both algae and the use of only one cycle was enough. A semi-purification process using Solid-phase Extraction (SPE) was carried out on both ASE crude extracts (one per species). The majority of phlorotannins were found in the ethanolic SPE fraction for *A. nodosum* and the hydroethanolic one for *H. siliquosa*. The SPE process allowed us to obtain more concentrated fractions of active phenolic compounds (×1.8 and 2 in *A. nodosum* and *H. siliquosa*, respectively). Results are discussed in regard to the exploitation of seaweeds in Brittany and to the research of sustainable processes to produce active natural ingredients for cosmetics.

## 1. Introduction

Seaweeds draw attention in various economic sectors for their particular properties: in France, 75% of the harvested seaweeds are used for the food-processing industry, chemistry, and microbiology and approximately 25% in agricultural, health and well-being sectors [1]. Living along marine coastal areas, seaweeds are affected by numerous biotic and abiotic environmental parameters that have an impact on their physiology, such as grazing by herbivorous (fish, molluscs), UV radiation or epiphytism [2,3,4,5,6]. They protect themselves against these stresses through the synthesis of specific molecules such as phenolic compounds, and then are the source of valuable compounds for several industrial sectors, particularly cosmetics [7,8]. In brown algae (Phaeophyceae), phenolic compounds are referred to as phlorotannins, which are phloroglucinol (1,3,5-trihydroxybenzene) units with different polymerization degrees [9]. Depending on the algal species, phlorotannin content can reach up to 25–30% DW and provide interesting biological functions as antimicrobial, antioxidant or photoprotective activities [7,10,11,12,13,14,15,16], and then can be used for various applications.

To be valorized, many steps such as the collection of pertinent samples, extraction, isolation of phlorotannins by purification or semi-purification processes and structural elucidation are required. Unfortunately, chemical procedures are often quite long and expensive, which hinders their industrial development. Even if traditional technologies with solvent extraction indicate a simple approach to metabolite isolation, there are several limitations such as low yield and purity, high solvent consumption and long processing times [17].

In this study, we were interested in developing a sustainable approach in order to isolate phlorotannins from brown seaweeds. As described by Murray et al. [18], the objective of a sustainable approach to producing active compounds from marine resources is to apply sustainable environmentally kind practices to the valorization of high-added-value biomolecules; these practices are carried out from the collection of samples, extraction and purification, to the obtaining of marine active ingredients.

Brittany, in the north-western part of France, is an area where the biomass and diversity of macroalgae is remarkable [19,20,21]. Among the high diversity of macroalgae in Brittany, we selected two different species, which synthetized different classes of phenolic compounds, *Ascophyllum nodosum* (Linnaeus) Le Jolis and *Halidrys siliquosa* (Linnaeus) Lyngbye which belong, respectively, to Fucaceae and Sargassaceae families [22,23,24,25]. Moreover, those two species are known to yield high phenolic content and/or active compounds [15,26,27].

In order to obtain phlorotannin extracts, green alternative methodologies and solvents are investigated to extract bioactive compounds by applying sustainable environmentally kind practices. Chemat et al. [28] defined green extraction as “all processes of extraction which reduce energy consumption, which allow alternative solvent and renewable products but also the insurance of a healthy and high-quality extract”. These authors have also defined a list of six principles for green extraction of natural products which should be consulted by industrial and scientific organisms to develop an innovative and green label. Many promising extraction processes can be found in the literature with Supercritical Fluid Extraction (SFE), micro-wave, enzymes and ultrasound-assisted extraction, and also Accelerated Solvent Extraction (ASE) [12,27,29,30,31,32,33,34]. This latter, also called Pressurized Fluid Extraction (PFE), Pressurized Liquid Extraction (PLE), Pressurized Solvent Extraction (PSE) or Enhanced Solvent Extraction (ESE) [35], is a green technology introduced in the 1990s, which is based on the same principle as traditional liquid extraction [36]. This technology is considered a green extraction because it is faster than a conventional extraction and uses small amounts of organic solvent [17,37]. Several publications have already highlighted its efficiency in the extraction of phenolic compounds from seaweeds [12,27,29,34,37,38,39]. A wide range of solvents can be used, giving the possibility to replace hazardous solvents with healthier ones for the environment such as water, ethanol or limonene. However, ASE is less selective than SFE because a wide range of compounds can be extracted simultaneously with this technique. Additional steps of purification must then be added to obtain specific molecules. For instance, Solid-phase Extraction (SPE) is a semi-purification method that allows compounds to be recovered through elution after their adsorption on a stationary phase contained in a cartridge [40]. This method was already used for the extraction of phlorotannins from seaweeds [27,41].

Also, in the context of sustainability, another objective was to find a method to rapidly monitor the presence/absence of phlorotannins in the purified extracts/fractions. Among the techniques allowing the visualization of phlorotannins, we chose proton Nuclear Magnetic Resonance spectroscopy (^1^H NMR), which has already proved its worth in numerous studies, for the quantification of phloroglucinol in the tissues of the brown alga *Ericaria selaginoides* (formerly *Cystoseira tamariscifolia*) [42], for obtaining chemical fingerprints for a taxonomic study of the genus *Turbinaria* [43,44], for the performance of a phlorotannins purification guidance in *Pelvetia canaliculata* [45], or for the detection of phenolic signals to compare different species of brown algae [46].

A focus on the ASE by varying different parameters has never been published on the two seaweeds of concern. The aims of this study were then (1) to propose the use of ^1^H NMR analysis to monitor the presence/absence of phenolic compounds in extracts/fractions to complement the time-consuming colorimetric test; (2) to propose ASE and SPE as environmentally friendly processes to extract and purify phenolic compounds. For this, parameters allowing the best, rapid and sustainable phenolic yield and activities using less algal powder, less solvent and non-toxic solvent, of these two sustainable technics, ASE and SPE, to, respectively, extract and purify phlorotannins, are researched; and, finally, (3) to show and compare radical scavenging, antioxidant and anti-aging activities of seaweed extracts obtained with ASE. We propose all these steps, ASE and SPE processes, followed by analysis using NMR as alternatives to classical extractive/purification/quantitative methods for industrial purposes in the cosmetics sector.

## 2. Results and Discussion

Our study dealt with the research of sustainable extraction and purification procedures in order to obtain active purified fractions of phlorotannins, which could be used in the cosmetics industry. For this, we studied the effect of (1) different extraction parameters of ASE (temperature and number of cycles) and (2) green solvents (water and ethanol) for the purification process on the phenolic content and associated bioactivities.

### 2.1. ^1^H NMR Spectra of ASE Extracts

**Figure 1:**
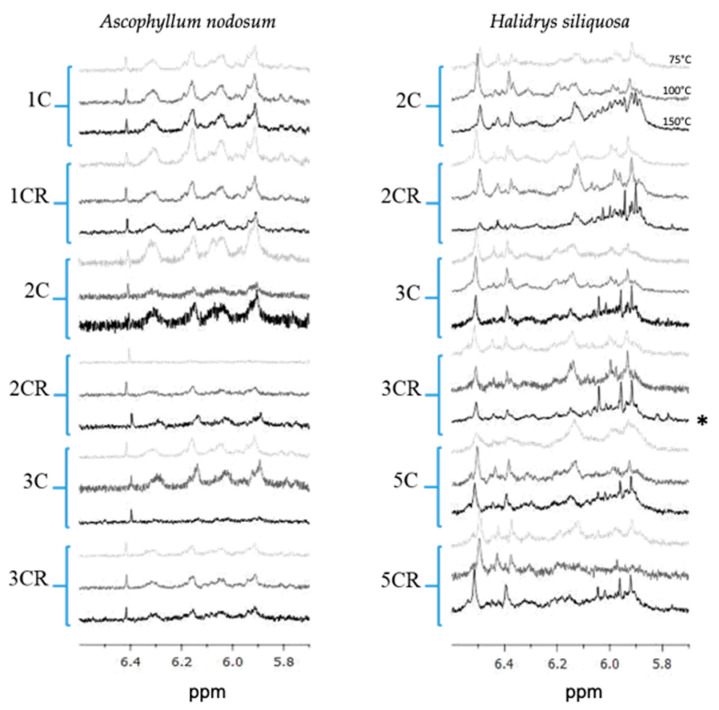
^1^H NMR analyses obtained on a Bruker Avance 400 and from samples dissolved within deuterated methanol. Focus on ASE extracts from *Ascophyllum nodosum* (**left**) and *Halidrys siliquosa* (**right**) in the 5.75–6.6 ppm area corresponding to phenolic compounds. The number (1C, 2C…) means the number of cycle(s) and “R” reflects cell inversion during the extraction cycles. The color of the spectra matches the temperature: light grey for 75 °C, dark grey for 100 °C and black for 150 °C. The spectrum with the highest total phenolic content (in Figure 2) is noted with a star *.

On ^1^H NMR spectra, we have selected only the zone where phenolic compounds are observed between 5.7 and 6.6 ppm (Figure 1).

In both algae, the presence of those molecules was observed in extracts, whatever the number of cycles and the temperature of the ASE extraction. The NMR signals present in the 5.75–6.6 ppm region are different between the two species, leading us to conclude that the phenolic profile between both species is different and that they do not produce the same phenolic compounds. The NMR signals present in the 5.75–6.6 ppm region are different between the two species, particularly in the region around 6 ppm where *H. siliquosa* presents massive signals, which is not the case for *Ascophyllum nodosum*, where the signals are more individualized. However, if we look precisely at the profiles for the different extraction conditions (number of cycles and temperature), the profile of *A. nodosum* was not modified whatever the condition, but the intensity of the peaks was lower when the number of cycles increased, while, with the latter, the *H. siliquosa* profile did not change with the number of cycles, but we can notice that the massive signals around 6 ppm increases with the extraction temperature, especially with the conditions 2C, 2CR and 3C (Figure 1).

### 2.2. Total Phenolic Content (TPC) and Extraction Yield Using ASE Process

**Figure 2:**
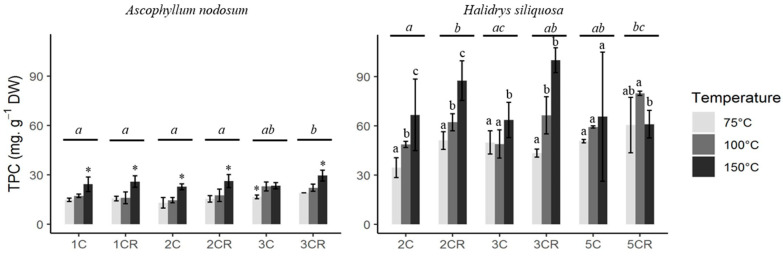
Total phenolic content (TPC) in extracts obtained by ASE with different cycles and temperatures (1C = 1 cycle, 1CR = 1 cycle with cell inversion) for *Ascophyllum nodosum* and *Halidrys siliquosa*. Stars and letters indicate differences between extracts according to temperature (stars; above the histograms) and according to cycles (letters; above the lines) (One-way ANOVA or Kruskal–Wallis, *p* < 0.05).

Extracts from *H. siliquosa* had a higher TPC than those from *A. nodosum*, with a maximum of 100.05 mg.g^−1^ algal DW and 29.51 mg.g^−1^ algal DW, respectively, for the same extraction procedure 3CR150 (3 cycles at 150 °C with the cell turned over; Figure 2). For both seaweeds, the temperature had a significant impact on the TPC (Kruskal–Wallis, *p* < 0.05). First, the highest TPC for each cycle was obtained at 150 °C for *A. nodosum* (One-way ANOVA, *p* < 0.05), except for the extract 3C (3 cycles), with no significant difference between 100 °C and 150 °C (One-way ANOVA, *p* = 0.833).

The highest TPC was also obtained at 150 °C for *H. siliquosa* for the extracts 2C, 2CR, and 3C (One-way ANOVA and Kruskal–Wallis, *p* < 0.05; Figure 2). Concerning the extract 3CR, no difference in TPC between 100 °C and 150 °C was highlighted (Kruskal–Wallis, *p* = 0.251) and no difference in TPC was shown with the extract 5C, whatever the temperature applied (Kruskal–Wallis, *p* = 0.814). For the extract 5CR, a significant difference in TPC was highlighted between 100 °C and 150 °C, with the highest level at 100 °C (Kruskal–Wallis, *p* = 0.041). The number of cycles did not affect TPC as much as the temperature for both seaweeds. Some significant differences were exhibited for the extract of *A. nodosum*: between 1CR and 2CR, 1CR and 3C and 3CR and 1C/2C/2CR (One-way ANOVA, *p* < 0.05). Regarding *H. siliquosa*, significant differences were found between 2CR and 2C/3C and 2C and 5CR (One-way ANOVA, *p* < 0.05). These differences between cycles did not follow a precise trend. The increase in the number of cycles or the implementation of the cell reversal did not increase the TPC for both seaweeds.

The number of cycles, each with a duration time of 5 min, increased the extraction time involving the extraction of different compounds or increasing the quantity of specific compounds. In the literature, one cycle with 20 min at 1500 psi was the extraction protocol currently used [12,47,48]. Moreover, Onofrejová et al. [49] decided to test phenolic extraction on the red macroalga *Pyropia tenera* (formerly *Porphyra tenera*) (Rhodophyta) and the brown macroalga *Undaria pinnatifida* using ASE with 3 cycles (5 min each) and a mixture of hexane/acetone to eliminate water insoluble compounds and other pigments, followed by another extraction with 80% methanol and 2 cycles (10 min each). The two-step elution was performed at 130 °C and 1885 psi. In another study, Zubia et al. [27] obtained ASE extracts from four species of Fucales, *Bifurcaria bifurcata*, *Ericaria selaginoides*, *Fucus ceranoides* and *Halidrys siliquosa* (Phaeophyceae) with 2 cycles (7 min each) at 75 °C and 1500 psi. Extraction time seems to be the factor having the weaker positive influence on the extraction of phenolic compounds compared to temperature. Indeed, our study showed that there was no significant difference in TPC and associated activities between the different cycles; therefore, we suggest the use of only one cycle because it is a shorter and less energetic process.

Concerning the extraction mass yield, it followed the trend of the TPC for both species: the highest yield was observed for *H. siliquosa* with 53.9 %, while *A. nodosum* had a maximum of 27.6% (always with the same procedure 3CR150; Table 1). When the temperature increased, the yield increased for most cycles, similarly to the TPC. Indeed, the highest yield was obtained at 150 °C for the extracts of *A. nodosum* (One-way ANOVA, *p* < 0.05), except for the extract 1CR, with no significant difference in yield between the three temperatures tested (One-way ANOVA, *p* = 0.052).

Regarding *H. siliquosa*, the highest mass yield was found at 150 °C for the extracts 2CR and 5C (One-way ANOVA, *p* < 0.05), while no difference of yield between 100 °C and 150 °C was exhibited for the extracts 3C and 3CR (One-way ANOVA, *p* > 0.05). Finally, no significant difference in mass yield was exhibited between temperatures for the extract 2C (Kruskal–Wallis, *p* = 0.270). Similarly to the TPC, the mass yield obtained for the extract with different numbers of cycles did not show a specific trend. The temperature and the number of cycles, which allowed us to obtain high TPC and yield, were 150 °C for both seaweeds and the smallest number of cycles, i.e., one cycle for *A. nodosum* and two cycles for *H. siliquosa*, because there was no effect of the number of cycles at the same temperature.

According to Kronholm et al. [50], extraction temperature is the major parameter influencing the physiochemical properties of solvent and extracted compounds, with high temperatures being generally more effective in increasing extraction yield. Indeed, Plaza et al. [47] highlighted that the higher the temperature is (between 50 °C, 100 °C, 150 °C and 200 °C), the higher the extraction yield for the brown macroalga *Himanthalia elongata*. Santoyo et al. [51] and Rodríguez-Meizoso et al. [52] showed also that the extraction yield from the microalga *Haematococcus lacustris* (formerly *H. pluvialis*) (Chlorophyta) increased with the extraction temperature (same temperatures as Plaza et al. [47]). In our study, extracts obtained at 150 °C compared to 75 °C and 100 °C, showed also the highest extraction yield. Tierney et al. [39] showed that extracts obtained through ASE produced a better yield than those obtained through solid–liquid extraction (SLE). Extraction time was shorter for ASE than SLE and temperature and pressure were higher for ASE than SLE causing the extraction of hydrophilic and also some less polar components [29,50,53]. In our study, the best extraction yield obtained for *A. nodosum* hydroethanolic extract (obtained with 3 cycles and cell inversion at 150 °C) was 27.6%, which was closer to the extraction mass yield of water extract generated at 120 °C and 1500 psi (28.7%) and higher than the hydroethanolic extract generated at 100 °C and 1000 psi (11.3%) of Tierney et al. [39]. However, the yield obtained at 100 °C with one or two cycles in our study agreed with this latter percentage: 13.1 and 13.0%, respectively. According to Del Pilar Sánchez-Camargo et al. [33], the extraction yield obtained for ASE extract after 20 min at 120 °C and 1500 psi for a Sargassaceae, *Sargassum muticum*, was 40.1%, which was a little weaker than our best results for the Sargassaceae, *H. siliquosa*, with 53.9% obtained after 3 cycles with the cell turn inversion at 150 °C.

The temperature can also impact the extraction of bioactive phenolic compounds from brown seaweeds, as discriminated by Sumampouw et al. [37] in *Fucus vesiculosus* and Getachew et al. [53] in their review. Tanniou et al. [12] observed that a temperature between 100 °C and 150 °C was better for yielding a high amount of phlorotannins from *Sargassum muticum,* without any degradation of phenolic signals using ^1^H NMR analysis, unlike SFE, which degraded phlorotannins signals. Several authors reported the sensitivity of phenolic compounds to temperature. In our study, thermosensitive compounds were apparently not degraded, as Richter et al. [36] suggested, because the highest TPC was obtained at 150 °C for both brown seaweeds. This phenomenon can be explained by an increase in the solubility of the compounds with temperature. Moreover, studies on higher plants showed no difference in TPC from rosemary between 150 °C and 200 °C, as the maximum amount of extractable phenolics was already reached at 150 °C [48]. In our study, the phlorotannin contents of *A. nodosum* extracts (between 80.4 and 132.0 mg.g^−1^ dry extract) were higher than hydroethanolic extract at 100 °C and 1000 psi (66.3 mg.g^−1^ of extract) obtained by Tierney et al. [39]. Temperature and pressure were similar between both studies. Another reason could explain this difference, like the harvesting sites of algae (i.e., Ireland for Tierney et al. [39] and Brittany for our present study) and environmental variations between sites, as it is known that they also have an impact on the phenolic content of seaweeds [11,54]. Zubia et al. [27] found that TPC of ASE extracts obtained with a mixture of dichloromethane and methanol at 75 °C and 1500 psi from *Halidrys siliquosa* was 16.0 mg.g^−1^ of DW, less than this study (between 34.6 and 60.6 mg.g^−1^ algal DW at 75 °C) but with a different solvent. Maybe the mixture using dichloromethane is not the most appropriate solvent to extract phenolic compounds [47]. Another Sargassaceae, *Sargassum muticum*, contained TPC close to 47.6 mg gallic acid equivalent GAE.g^−1^ extract obtained after ASE at 120 °C and 1500 psi [33], which was lower than our best result obtained for *H. siliquosa* (165.0 mg.g^−1^ of extract). However, their results were expressed in gallic acid equivalent, which is better found in green and red seaweeds, and which does not correspond to the monomer structuring phlorotannins in brown seaweeds [9]. The use of this different phenolic standard could have an effect on the seaweed TPC calculated from the standard curve from one or the other phenolic compound.

### 2.3. Antioxidant Activities of ASE Extracts

**Figure 3:**
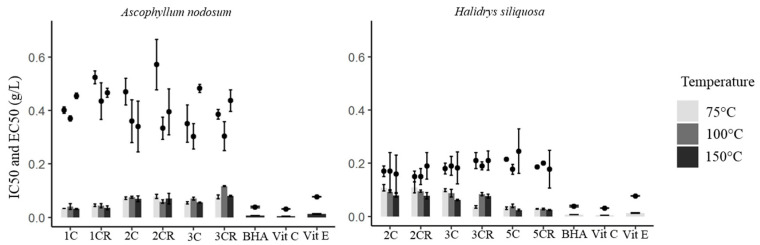
Radical scavenging and antioxidant activities from two different colorimetric tests, DPPH (IC50; points) and FRAP (EC50; histograms) of extracts obtained by ASE with different cycles (1C = 1 cycle, 1CR = 1 cycle with cell inversion, 2C = 2 cycles, etc.) and temperatures (75 °C, 100 °C and 150 °C) for *Ascophyllum nodosum* (**left**) and *Halidrys siliquosa* (**right**). Statistical results are shown in Supplementary data.

According to the DPPH test, *H. siliquosa* showed better radical scavenging activities than *A. nodosum*, with the most interesting concentration being 0.15 mg.mL^−1^ for the former and 0.30 mg.mL^−1^ for the latter (Figure 3). Moreover, the IC50 of the extracts from *H. siliquosa*, varying from 0.15 to 0.22 mg.mL^−1^, were close to some positive controls (vitamins C, E and BHA) with generally no significant difference in activities (Kruskal–Wallis, *p* > 0.05). For *A. nodosum*, the extracts were less active than positive controls. On the contrary, for TPC and mass yield, the temperature did not affect as much the radical scavenging activity determined by the DPPH test. For *A. nodosum*, only a significant difference was highlighted between 100 °C and 150 °C for the extract 2CR with the highest radical scavenging activity obtained at 150 °C (Kruskal–Wallis, *p* = 0.031). The temperature had no impact on radical scavenging activities.

Similarly to TPC and mass yield, and considering the results of the DDPH test, the number of cycles did not influence a specific trend in the radical scavenging activities. However, significant differences in the reducing activity (FRAP) were highlighted between the extract 1CR and the extracts 2C, 3C and 3CR, suggesting that a low number of cycles allowed high radical scavenging activities in *A. nodosum*.

Our results showed then that the temperature had a lower impact on the radical scavenging activity determined by DPPH compared to TPC. Herrero et al. [48] showed that the activity by the DPPH method increased with an increase in temperature up to 200 °C on rosemary plants. Another study showed higher antioxidant activities of *Himanthalia elongata* extracts obtained when water temperature increased from 50 °C to 200 °C; meanwhile, ethanol extracts showed their maximum at 100 °C compared to 50 °C, 150 °C or 200 °C [47]. Between our extracts obtained with different cycle numbers, some showed a more interesting antioxidant activity at 150 °C or even 100 °C compared to 75 °C. Otherwise, no significant difference was observed. If we compared our results with the literature, our radical scavenging activities were better or closer to theirs. Tanniou et al. [12] showed in *Sargassum muticum* extracts obtained at 120 °C and 1500 psi, an IC50 of 0.77 mg.mL^−1^ with DPPH test, which was less active than our results obtained for another Sargassaceae *H. siliquosa* with 0.15 mg.mL^−1^. In another study, Zubia et al. [27] showed an IC50 close to our results with 0.21 mg.mL^−1^ (at 75 °C and 1500 psi). Between our two seaweed models, *H. siliquosa* extracts presented better radical scavenging activities.

According to the FRAP test, profiles between both seaweeds were different compared to the previous test (Figure 3). For *A. nodosum*, the EC50 was closer to the positive controls with the extract 1C, rather than the extract 5CR of *H. siliquosa* with 0.032 mg.mL^−1^ at 75 °C and 0.024 mg.mL^−1^ at 150 °C, respectively. As for the radical scavenging activity, the temperature did not affect the antioxidant activity determined by FRAP assay for the extracts 1C, 1CR and 2C for *A. nodosum*. However, significant differences were found between 100 °C and 150 °C for the extracts 2CR, 3 and 3CR (Kruskal–Wallis, *p* = 0.001, *p* = 0.003 and *p* < 0.001, respectively), with the highest antioxidant activity obtained at 150 °C for the extracts 3C and 3CR, and at 100 °C for the extract 2CR. For *H. siliquosa*, the temperature did not affect the antioxidant activity for the extracts 2C and 5CR. However, differences in antioxidant activity were shown between 75 and 150 °C for the extracts 2CR, 3C and 3CR (Kruskal–Wallis, *p* = 0.004, *p* = 0.030 and *p* = 0.002, respectively), with the highest antioxidant activity obtained at 150 °C for the extracts 2CR and 3C, and 75 °C for the extract 3CR. A significant difference was also highlighted between 75 °C and 100 °C for the extract 3CR and between 100 °C and 150 °C for the extract 5C, with the highest antioxidant activity obtained at 75 °C and 150 °C, respectively (Kruskal–Wallis, *p* < 0.001 for both). Finally, the temperature did not have a real impact on activities and allowed the highest antioxidant activities at 150 °C. About the effect of the number of cycles on the antioxidant activity determined by FRAP assay, for *A. nodosum*, the extracts 1C and 1CR were significantly different from the extracts 2C, 2CR, 3C and 3CR (Kruskal–Wallis, *p* < 0.001) with better activities with the lowest number of cycles. Conversely, the extracts 2C and 2CR of *H. siliquosa* were significantly different from the extracts 3CR, 5C and 5CR with better activities with the highest number of cycles (Kruskal–Wallis, *p* < 0.01). In the same way, a significant difference was found between 3C and 5C, and 3CR and 5C/5CR (Kruskal–Wallis, *p* < 0.002). According to the antioxidant activity determined by the FRAP assay, the results showed that the temperature had a lower impact as already demonstrated for the radical scavenging activity. However, the most interesting reducing activity was found at 150 °C for some extracts of *A. nodosum* (2CR and 3C) and *H. siliquosa* (3C and 3CR). High reducing activity of the extract of *H. siliquosa* obtained at 75 °C, 1500 psi and a mixture of dichloromethane/methanol has already been observed [27]. These authors found 72.4% inhibition at 0.005 mg.mL^−1^, which corresponds to an EC50 of around 0.003 mg.mL^−1^, which is much more active than our best extract showing 0.023 mg.mL^−1^. They also found interesting activities for two Fucaceae (same family of *A. nodosum*), *Fucus ceranoides* and *F. serratus*: 66.0 and 43.7% at 0.02 mg.mL^−1^, which correspond to EC50 of 0.015 and 0.023 mg.mL^−1^, respectively. Those results are in agreement with ours obtained for the best extract of *A. nodosum* with an EC50 of 0.023 mg.mL^−1^.

### 2.4. Photoprotective Sunscreen Activities of ASE Extracts

The extracts from *A. nodosum* showed no photoprotective activity. As shown in Figure 2, *A. nodosum* has a lower phenolic content than *H. siliquosa*. As a result, the radical scavenging and antioxidant activities (Figure 3) are lower than with *Halidrys* and the phenolic compounds detected in *Ascophyllum* are too low to allow sufficient (significant) absorption of UV radiation. We were therefore unable to obtain SPF (Sun Protection Factor) values significantly different from the control. Moreover, *Ascophyllum* was harvested in January, at a time when there is not much sunshine, unlike the *Halidrys* harvesting period in April, when there is more sunshine. We can therefore hypothesize that *Ascophyllum* does not develop photoprotection during its harvesting period (January), whereas *Halidrys*, harvested in April, develops a photoprotection strategy by producing more PC.

In the present study, only the *H. siliquosa* results of SPF are presented (Table 2). Similarly to TPC and mass yield, the highest SPF and PF-UVA for *H. siliquosa* was obtained at 150 °C and was significantly different from the values at 75 °C and 100 °C (Kruskal–Wallis, *p* < 0.05), except for 2CR with no difference in SPF and PF-UVA between temperatures (Kruskal–Wallis, *p* = 0.071 and *p* = 0.061, respectively) (Table 2). The highest SPF and PF-UVA were 2.33 and 1.85, respectively, for the extract 2C150. No significant difference was observed between the values of SPF and PF-UVA obtained at 75 °C and 100 °C whatever the number of cycles, except for the extract 5CR (Kruskal–Wallis, *p* = 0.043 for both).

In our study, the temperature of 150 °C allowed us to obtain the highest SPF and PF-UVA for *H. siliquosa* with 2.33 and 1.85, respectively. These results were closer to those obtained by Le Lann et al. [15] with a SPF of 3.55 and PF-UVA value of 2.20 for the phlorotannin-enriched fraction of *H. siliquosa*. Our values were close to another solar organic filter like homosalate which presents a SPF greater than 4 [55]. 

### 2.5. Anti-Aging Activities of ASE Extracts

Anti-aging activities were evaluated by elastase and tyrosinase inhibitions on six extracts obtained by ASE: 1C at 75 °C, 100 °C and 150 °C for *A. nodosum* and 2C at 75 °C, 100 °C and 150 °C for *H. siliquosa*. The anti-elastase activity of extracts of *A. nodosum* was better than the positive control, EGCG (Table 3), at the same concentration. However, the extracts showed less tyrosinase inhibition than the positive control, kojic acid. Regarding the anti-tyrosinase activity, *H. siliquosa* extracts showed less activity than extracts from *A. nodosum*, while a higher tyrosinase inhibition was shown at 75 °C and 150 °C. Compared to the positive controls, extracts from *H. siliquosa* were almost similar for anti-elastase activity and less active for anti-tyrosinase activity.

Extracts from both seaweeds exhibited anti-aging potentials with anti-elastase and anti-tyrosinase activities. According to the elastase inhibition, the activity was even higher than the positive control for *A. nodosum* but not for *H. siliquosa*. A previous study found anti-elastase activity of an extract of *Sargassum muticum* obtained after enzyme-assisted extraction [56]: authors showed between 21.6 and 32.8% of elastase inhibition at 1 mg.mL^−1^, which was less than our results, between 47.4 and 80.5%.

Both seaweed extracts have then interesting valorization potential for their different biological activities such as antioxidant and anti-aging activities, with photoprotective activity found only in *H. siliquosa*. The temperature appeared to have an impact but not for all extracts. However, the temperature of 150 °C was largely dominant for obtaining the best activities.

### 2.6. Correlation between Phenolic Compounds and Activities for ASE Extracts

**Figure 4:**
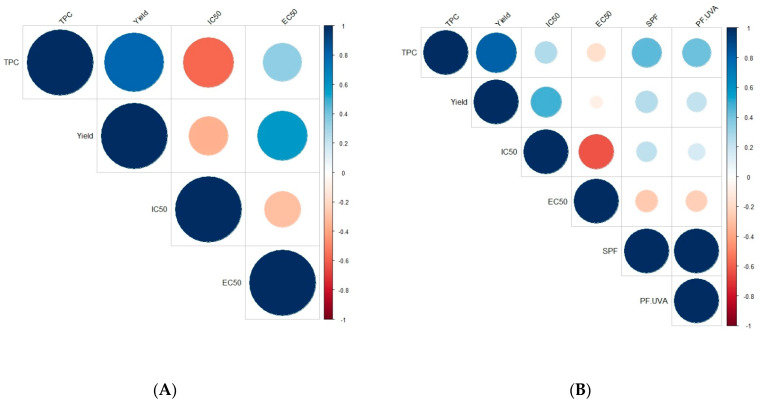
Correlations between TPC, mass yield, radical scavenging activities (DPPH = IC50), antioxidant activities (FRAP = EC50) for *Ascophyllum nodosum* (**A**) and *Halidrys siliquosa* (**B**) and photoprotective activities (SPF and PF-UVA) for *Halidrys siliquosa* (**B**). Positive correlations are displayed in blue and negative correlations in red. The intensity of the color and the size of the circles are proportional to the correlation coefficients (Pearson’s correlation).

As shown in Figure 4, three positive correlations were found for *A. nodosum* with only two that were statistically significant: the strongest correlation was observed between TPC and extraction mass yield (r = +0.794, *p* < 0.001), followed by the correlation between the yield and the antioxidant activity determined by the FRAP assay (EC50; r = +0.573, *p* = 0.013) (Figure 4). In contrast, three negative correlations were noticed with only one that was statistically significant: between the TPC and the radical scavenging activity—DPPH assay (IC50; r = −0.579, *p* = 0.012). For *H. siliquosa*, nine positive and six negative correlations were found with only four statistically significant (Figure 4). The strongest positive (and expected) correlation was obtained between the two photoprotective activities, SPF and PF-UVA, with an r coefficient equal to +0.991 (*p* < 0.001). Similarly to *A. nodosum*, a strong positive correlation was highlighted between TPC and mass yield (r = +0.817, *p* < 0.001). Two other significant correlations were observed between the yield and the radical scavenging activity (r = +0.481, *p* = 0.043), and between the two antioxidant activities (r = −0.624, *p* = 0.006).

In our study, phenolic content was strongly correlated to the extraction mass yield showing that the extraction was well specific to phenolic compounds. Phenolic compounds could also be responsible for biological activities. In our study, a negative correlation was highlighted between TPC and radical scavenging activity (expressed as IC50) for *H. siliquosa* or between mass yield and radical scavenging activity for *A. nodosum*. This means that the higher the content or the yield was, the lower the IC50 and therefore the better the activity, as already demonstrated by Balboa et al. [57] in seaweeds. Moreover, according to Audibert et al. [58], radical scavenging activity was correlated with the content of phenolic compounds for *A. nodosum* fractions obtained by ultrafiltration. Other studies found some negative correlations between the phenolic content and the radical scavenging activity (or the opposite) on crude extracts of brown seaweeds: in *Ecklonia cava* subsp. *stolonifera* (formerly *E. stolonifera*) [59], *Himanthalia elongata* and *Laminaria* sp. [60], *Sargassum* sp. [61], *Ericaria sedoides* (formerly *Cystoseira sedoides*) [62], *Fucus serratus* and *Ascophyllum nodosum* [63] or *Fucus vesiculosus* [63,64]. Depending on the species of concern, no correlation was found depending on the way of extraction or the solvent used for example. Indeed, Zubia et al. [27] did not find any correlation between the total phenolic content and the radical scavenging and reducing activities of crude extracts from the Fucales *Ericaria selaginoides* or *Fucus ceranoides*. On the contrary, they found this correlation on SPE purified fractions and in other brown seaweeds: *Bifurcaria bifurcata, Ericaria selaginoides, Fucus ceranoides* and *Halidrys siliquosa* [27]. Other studies also found this correlation between TPC and reducing activity: in the crude extract of the Laminariales *Ecklonia cava subsp. stolonifera* with a r coefficient equal to +0.963 [59], and also for the purified extract of *Halidrys siliquosa* [15], given the similarity of chemistry between both tests (Folin–Ciocalteu quantification and FRAP test), as described by Huang et al. [65]. 

On the other hand, in our study, a negative correlation between radical scavenging activity and reducing activity (FRAP) was highlighted, as already demonstrated by Zubia et al. [27] for crude extracts of Fucales, i.e., *Ericaria selaginoides*, *B. bifurcata* and *F. ceranoides*. However, as those three assays (FC, DPPH and FRAP) rely on a mechanism of electron transfer [65], maybe some other compounds present in the extracts are responsible for this opposite behavior. This last behavior could also be due to the different pH values of these assays, i.e., acidic (FRAP) and basic (FC) conditions, as described by Huang et al. [65]. In their work, these last authors demonstrated protonation or proton dissociation on antioxidant compounds, depending on the pH of the solution [65].

### 2.7. Semi-Purification of ASE Extracts Using SPE Procedure

**Figure 5:**
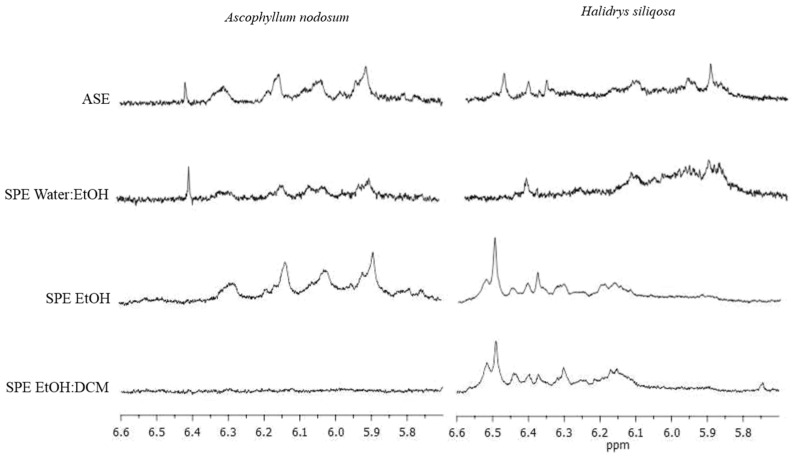
^1^H NMR profiles obtained on a Bruker Avance 400 and from samples dissolved within deuterated methanol. Focus on the zone of phenolic compounds between 5.7 and 6.6 ppm, of SPE fractions carried out on ASE extracts 1C150 for *Ascophyllum nodosum* (on the left) and 2C150 for *Halidrys siliquosa* (on the right).

The SPE semi-purification procedure was carried out on two extracts obtained by ASE: 1C150 for *A. nodosum* and 2C150 for *H. siliquosa,* given that these extracts brought together the highest total phenolic content (TPC), the best antioxidant and photoprotective activities. The signal of phenolic compounds on ^1^H NMR analyses was found on two fractions for *A. nodosum*, hydroethanolic (Water/EtOH) and ethanolic (EtOH) fractions, and three fractions for *H. siliquosa*, hydroethanolic, ethanolic and dicholoromethane–ethanolic (EtOH/DCM) fractions (Figure 5).

**Figure 6:**
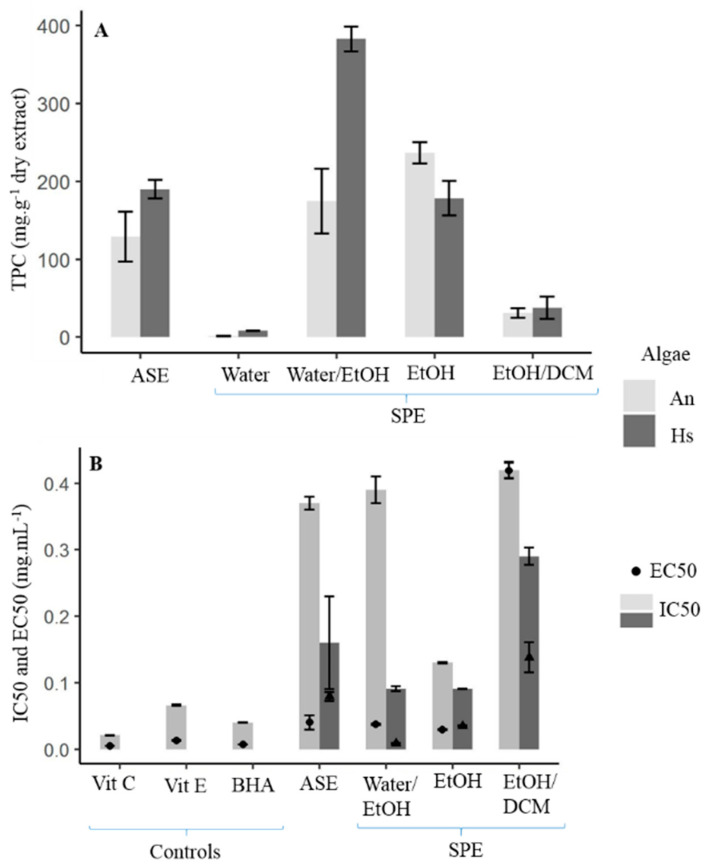
Total phenolic content (TPC) (**A**), radical scavenging activities (DPPH, IC50, bars) and antioxidant activities (FRAP, EC50, points) (**B**) in fractions obtained by SPE from ASE extracts of *Ascophyllum nodosum* and *Halidrys siliquosa*. See statistical results in Supplementary Data (Table S1). Vit C: Vitamin C (=ascorbic acid), Vit E: Vitamin E (=α-tocopherol), BHA: Butylated hydroxyanisole, ASE: Accelerated Solvent Extraction, EtOH: Ethanol, DCM: Dichloromethane.

For *A. nodosum*, the shape profile was almost similar on both fractions and a signal at 6.4 ppm was only present for the Water/EtOH fraction (Figure 5). This peak was also found on the profile of the ASE crude extract (Figure 1). The NMR profiles of *H. siliquosa* SPE fractions were different: like for *A. nodosum*, the Water/EtOH fraction looked like ASE extract. However, the shape profile was completely different for the two other fractions but similar to each other. In Figure 6, the TPC was expressed as mg.g^−1^ of the dry fraction, which allows us to show, or not, the concentration of phenolics compared to the crude ASE extract.

For *Ascophyllum nodosum*, the highest TPC was found within the EtOH fraction with 237 mg.g^−1^ of dry fraction while for *H. siliquosa* it was the Water/EtOH fraction with 383 mg.g^−1^ of dry fraction (Figure 6A). The semi-purification, based on the use of the SPE procedure, allowed them to concentrate the phenolic compounds by 1.8 for *A. nodosum* and 2 for *H. siliquosa*.

The radical scavenging activities according to the DPPH test were closer to the positive controls for the EtOH fraction of *A. nodosum* (0.13 mg.mL^−1^) and the Water/EtOH and EtOH fractions of *H. siliquosa* (0.09 mg.mL^−1^ for both; Figure 6B). Even if activities were not significantly different (Kruskal–Wallis, *p* > 0.05), the EtOH fraction of *A. nodosum* appeared to have a better radical scavenging activity than its ASE extract (0.37 mg.mL^−1^). The difference between the extract and the semi-purified fractions was not as strong with *H. siliquosa*.

According to the FRAP test, Water/EtOH and EtOH fractions from *A. nodosum* and *H. siliquosa* were close to positive controls (vitamins C and E, BHA) with 0.04 and 0.03 mg.mL^−1^ for the first seaweed, and 0.008 and 0.03 mg.mL^−1^ for the second seaweed, respectively. For both species, values of antioxidant activity for the SPE fractions were not statistically different except between Water and Water/EtOH fractions for *H. siliquosa* with the highest antioxidant activity for the Water/EtOH fraction (Kruskal–Wallis, *p* = 0.028).

Semi-purification made it possible to obtain fractions with photoprotective activity for *A. nodosum*, whereas there was no activity for its crude ASE extract. Indeed, the EtOH fraction showed SPF and PF-UVA of 1.42 and 1.29, respectively (Table S2, Supplementary Data). However, regarding *H. siliquosa*, enriched fractions (Water/EtOH and EtOH fractions) had significantly lower SPF and PF-UVA compared to the crude ASE extract (Kruskal–Wallis, *p* < 0.005). 

Semi-purification by Solid-phase Extraction (SPE) allowed us to obtain a higher TPC and better reducing activities for both seaweeds, compared with the crude ASE extract. For *H. siliquosa* NMR spectra, different signals observed with the different solvents could be explained by the hypothesis that different structures of phlorotannins were obtained: those more hydrophilic, which were extracted with the mixture of water and ethanol, and those with a structure more hydrophobic extracted with 100% ethanol and the mixture of ethanol and dichloromethane. Many other molecules were eliminated by SPE: for example, lipids were removed from the hydroethanolic fraction and found in the ethanolic fraction and dichloromethane–ethanolic fractions (Supplementary Data, Figure S1). The SPE procedure allowed them to concentrate fractions into phenolic compounds. Indeed, the phenolic content was multiplied by 2 thanks to the SPE process for the Water/EtOH fraction of *H. siliquosa* and by 1.8 for the EtOH fraction of *A. nodosum*. Zubia et al. [27] also used SPE on dichloromethane/methanol ASE extract to concentrate phenolic compounds. For four brown seaweeds, these authors showed this concentration: 1.0% of dry weight (DW) for the ASE crude extract and 13.3% DW for the SPE water fraction of *Bifurcaria bifurcata*; 5.5% DW for the ASE crude extract and 41.9% DW for the SPE hydromethanolic fraction of *Fucus ceranoides*; 10.9% DW for the ASE crude extract and 25.4% DW for the SPE water fraction of *Ericaria selaginoides*; and 1.6% DW for the ASE crude extract and 28.8% DW for the SPE hydromethanolic fraction of *Halidrys siliquosa*. Rajauria [66] showed also a concentration of phenolics by 1.6 between a crude methanolic extract obtained by maceration and a SPE methanolic fraction of *Himanthalia elongata* with 178.2 and 279.5 mg GAE.g^−1^, respectively. They used this SPE process to concentrate phenolic compounds and that allowed them to identify further phlorotannins in the fraction by HPLC-MS.

For the antioxidant activities found in our study, even if SPE fractions showed better activities for both assays, they were not significantly different from the crude ASE extract. However, activities of Water/EtOH and EtOH fractions from *H. siliquosa* and EtOH fraction from *A. nodosum* were similar to positive controls, i.e., BHA and vitamins C and E. One should hypothesize that only apolar phlorotannins are active in *A. nodosum* while both polar and apolar phlorotannins are active in *H. siliquosa*. In the literature, Onofrejová et al. [49] have found that antioxidant activities were similar between ASE extract and SPE fractions for the Laminariales *Undaria pinnatifida*. On the contrary, Rajauria [66] found a better antioxidant capacity (EC50) for the SPE methanolic fraction of *H. elongata* compared to the crude extract with 14.5 and 46.3 μg.mL^−1^, respectively. This SPE fraction was also better than the positive control, vitamin C.

Finally, and interestingly, in our study, photoprotective activity appeared for the SPE fraction of *A. nodosum*; meanwhile, this activity decreased for *H. siliquosa*. The photoprotective activity could maybe be due to a type of phlorotannins or is the result of a complexation of phlorotannins with other molecules in the crude extract.

### 2.8. Interest in Using NMR to Check the Presence/Absence of Phlorotannins in Extracts

In order to quantify phlorotannins, a colorimetric test using the Folin–Ciocalteu procedure is normally used in numerous studies in reference to a phloroglucinol standard range. In the present study, we propose to complement the FC procedure by using ^1^H NMR spectra in order to check the presence/absence of phlorotannins, i.e., aromatic signals, in order in order to save time. In previous work, qNMR was used to quantify phlorotannins in the brown macroalga *Ericaria selaginoides*, as this species produces the monomer phloroglucinol [42], identified by the presence of a singlet at 6 ppm on a ^1^H NMR spectrum. Unfortunately, both species of concern here, *Ascophyllum nodosum* and *Halidrys siliquosa*, do not produce the monomer phloroglucinol but rather polymerized phlorotannins, as already demonstrated by previous studies [15,25,67]. Although Wekre et al. [68] demonstrated the possibility of ^1^H NMR for quantifying polyphenols in brown algae, in our case, it was not possible to quantify phlorotannins in the two species studied. The ^1^H NMR analysis thus allows us to detect the presence of phlorotannins by signals present between 5.5 and 6.5 ppm, and to deduce the relative proportion of phlorotannins in the sample, visible by the height and the form of the signals, which agree with the contents of phenolic compounds determined by the colorimetric test (Folin–Ciocalteu procedure). Moreover, it also makes it possible to appreciate the complexity of polymers. Indeed, species producing the monomer phloroglucinol will present a singlet at 6 ppm [42], while species producing more complex phlorotannins will present a high number of peaks, a sign of a very heterogeneous structure of phlorotannins with ramifications.

## 3. Materials and Methods

### 3.1. Algal Material

Two brown seaweeds (Ochrophyta, Phaeophyceae, Fucales), *Ascophyllum nodosum* (Linnaeus) Le Jolis (Fucaceae) and *Halidrys siliquosa* (Linnaeus) Lyngbye (Sargassaceae) were collected in Cotes d’Armor (Brittany Region, France) by a professional harvester and dried by the SME C-WEED Aquaculture, in ventilated heating conditions, with a temperature below 40 °C. The sampling collection was made in January 2016 for *H. siliquosa* and in April 2016 for *A. nodosum*, with the harvest of non-mature thalli in both species. Seaweeds were collected in Brittany with respect to the preservation of seaweed fields and the life cycle of the alga and were dried and then ground to powder using a ball mill (Retsch MM400, Haan-Gruiten, Germany). We selected two different species that produce different types of phlorotannins, as visible in the phlorotannins overview in Fucales made by Catarino et al. [25].

### 3.2. Extraction and Purification Procedures

**Accelerated Solvent Extraction (ASE):** Extractions were performed thanks to an Accelerated Solvent Extractor system (Dionex^TM^ ASE^TM^ 150, Sunnyvale, CA, USA). In all cases, i.e., species and conditions, 3 g of dried algae mixed with sand were loaded into a 10 mL stainless steel extraction cell, fitted with glass-fiber filters at the inlet and outlet. Extraction parameters were chosen according to earlier studies [12,27,33,34,39,51,69]. Mixtures composed of water and ethanol were used with a ratio of 75:25, as described by Tanniou et al. [12] for Sargassaceae and Ford et al. [70] in *Ascophyllum nodosum*. Static time (the time the solvent remains in the cell) was 5 min (time of one cycle) and the pressure was set at 100 bar (=1500 psi). Extractions were performed with different numbers of cycles (1, 2, 3 or 5, with the cell turned over or not) at three different temperatures (75 °C, 100 °C and 150 °C). The applied rinse volume was 100% and the purge time was 120 s. After extraction, the solvent was evaporated using a rotary evaporator (Laborota 4000 efficient, Heidolph, Germany) and the extract was freeze-dried (Beta 1–8 LD, Christ, Osterode am Harz, Germany).

**Semi-purification by Solid-phase Extraction (SPE).** ASE extracts showing the highest total phenolic content (TPC) and the best antioxidant and photoprotective activities were selected to realize a semi-purification using a Solid-phase Extraction (Vac Elut SPS 24, Agilent Technologies, Santa Clara, CA, USA). The compounds in the liquid phase were separated from the other elements by selective adsorption on a solid phase according to physicochemical properties. Ready-to-use SPE cartridges containing C18 silica (Strata C18-E, 55 μm, 70, 20 g/60 mL, Giga Tubes, Phenomenex, Torrance, CA, USA) were used. The SPE cartridge was first primed with ethanol and distilled water and followed by a deposit of 0.5 g of the crude extract mixed with silica. Solvents with different polarities were added to the cartridge to obtain five fractions: distilled water, distilled water/ethanol (50:50), ethanol, ethanol/dichloromethane (50:50) and dichloromethane. In order to maximize the contact between the extract and the different solvents, the solvents were passed through the cartridge four times. The solvents were then evaporated using a rotary evaporator (Laborota 4000 efficient, Heidolph, Schwabach, Germany) and fractions were freeze-dried (Beta 1–8 LD, Christ, Germany).

### 3.3. Quantification of Phenolic Compounds

**Total phenolic content (TPC) using the Folin–Ciocalteu procedure.** The TPC was colorimetrically determined by spectrophotometry using an adapted Folin–Ciocalteu assay [27]. The wells were filled with 20 μL of extract, 130 μL of distilled water and 10 μL of Folin–Ciocalteu reagent, followed by 40 μL of Na_2_CO_3_ (200 g.L^−1^). The mixture remained for 10 min at 70 °C. The microplate was then put on ice to stop the chemical reaction and the absorbance was read at 620 nm (Multiskan^TM^ FC, Thermo Fisher Scientific^TM^, Waltham, MA, USA). The TPC was expressed in milligrams per gram of algal dry weight (mg g^−1^ DW) or per gram of extract (or fraction) dry weight from a standard curve of phloroglucinol (1,3,5-trihydroxybenzene). This analysis was carried out in triplicate for each extract.

**^1^H Nuclear Magnetic Resonance (NMR) analysis of extracts**: Molecular signatures of extracts and fractions were obtained using ^1^H NMR analyses using a Bruker Avance 400. For each analysis, 5 mg of extract was dissolved in 700 μL of deuterated methanol (MeOD). The spectra were analyzed with MestReNova software (v6.0.2-5475). ^1^H NMR spectra gave information on the composition of extracts and especially the signal of phenolic compounds, which appeared between 6.5 and 5.5 ppm.

### 3.4. Activity Tests 

**DPPH radical scavenging assay:** The 2,2-diphenyl-1-picrylhydrazyl (DPPH) radical scavenging assay modified according to Zubia et al. [27] was used to determine the radical scavenging activity of extracts and fractions. For this method, 22 μL of extracts/fractions was added to 200 μL of DPPH solution (25 mg.L^−1^), prepared fresh daily. After 60 min in the dark at room temperature, absorbance was read at 540 nm. Distilled water was used as a negative control and ascorbic acid (vitamin C), α-tocopherol (vitamin E), together with 2,3-t-butyl-4- hydroxyanisole (butylated hydroxyl-anisole, BHA) and 2,6-di-tert-butyl-4-methylphenol (butylated hydroxytoluene, BHT) as positive controls. This analysis was carried out in triplicate for each extract/fraction. The radical scavenging activity of extracts/fractions was expressed as IC50 (mg.mL^−1^), the concentration of substrate that causes a 50% loss of the DPPH activity. A weak IC50 is indicative of a high antioxidant activity.

**Ferric reducing antioxidant power (FRAP):** Reducing activity was evaluated by the reaction of oxydoreduction between phenolic compounds of the extract/fraction and transition metal ions like ferric irons. The method used was adapted from Zubia et al. [27]. On the microplate, 25 μL of sodium phosphate buffer (0.2 M, pH 6.6) and 25 μL of ferricyanide potassium at 1% were added to 25 μL of extracts/fractions (or controls). After homogenization, microplates were incubated at 50 °C for 20 min. The reaction was stopped on ice. A first measurement of the absorbance at 620 nm was made after the addition of 25 μL of trichloroacetic acid and 100 μL of distilled water. Finally, 20 μL of iron chloride was added. After 10 min, absorbance was measured at 620 nm. BHA, BHT, and vitamins C and E were used as positive controls. This analysis was carried out in triplicate for each extract/fraction. The ferric reducing antioxidant power was expressed as the EC50 value (mg.mL^−1^) after calculating a percentage of inhibition through comparison to a blank (distilled water). A lower EC50 value means a high reducing power of the sample.

**Elastase inhibition:** Elastase degrades elastin, which is responsible for skin elasticity. An anti-elastase activity therefore prevents a loss of elasticity and acts as an anti-aging agent. The protocol was inspired by Sallenave et al. [71]. First, a Tris-HCl buffer was made at a concentration of 179 mM and a pH of 8. The substrate, N-Succinyl-Ala-Ala-Pro-Phe p- nitroanilide, was then prepared at 1.65 mM with the buffer. The last solution was the enzyme diluted with the buffer to obtain a concentration of 0.34 U.mL^−1^. On the microplate, 13 μL of extract or control were added with 93 μL of buffer and 52 μL of substrate. The microplate was incubated for 5 min at 25 °C. Then, 42 μL of enzymes was finally added. A kinetic was performed at 410 nm, always at 25 °C for 4 min. Epigallocatechin gallate (EGCG) was used as a positive control. To obtain the percentage of inhibition of the enzyme, the slope, which corresponds to the initial speed of the enzyme, was calculated and used in the following equation:

% inhibition = ((Si − SiF)/Si) × 100

with Si, the initial speed of the enzyme without the extract or the control, and SiF, the initial speed of the enzyme with the extract or the control. Only ASE extracts showing the highest total phenolic content (TPC) were selected to determine their anti-elastase activity.

**Tyrosinase inhibition:** Depigmentation considered an anti-aging activity was evaluated thanks to the assessment of the tyrosinase inhibition activity of extracts. The protocol was inspired by Chan et al. [72] and Lim et al. [73]. A phosphate buffer was prepared at pH 6.5 and 0.1 M by mixing 4.117 g of Na_2_HPO_4_ and 8.639 g of NaH_2_PO_4_. The second solution, corresponding to the substrate, consists of a 1.694 mM L-tyrosine solution solubilized in distilled water. A volume of 2.5 mL of this solution was then added to 2.5 mL of phosphate buffer and 2.25 mL of distilled water to obtain the L-tyrosine reagent. Then, the enzymatic solution was at a concentration of 317.71 U.mL^−1^ of tyrosinase. In the microplate, 20 μL of extract or control were added with 243 μL of L-tyrosine reagent and the mixture was incubated at 25 °C for 5 min. Finally, 42 μL of enzymes were added and a kinetic was performed at 475 nm, always at 25 °C, for 5 min. A blank was made for each extract replacing substrate with buffer. Kojic acid was used as a positive control. The percentage of inhibition of the enzyme was calculated in the same way as for the elastase inhibition.

**Photoprotective efficacity:** The Sun Protection Factor (SPF and PF-UVA) was determined to obtain the photoprotective efficiency of extracts and UV-B and UV-A ranges. The method is described by Couteau et al. [55,74]. Briefly, the extract was incorporated in a basic oil-in-water (O/W) emulsion and 50 mg was spread with the finger on a polymethylmethacrylate (PMMA) plate. Then, transmission measurements were made on 9 replicates using a spectrophotometer (UV transmittance Analyzer UV1000S, Labsphere, North Sutton, NH, USA).

### 3.5. Statistical Analyses

The software R for Windows was used with the RStudio (v.1.0.136) integrated development environment. All laboratory analyses were performed in triplicate, and results were expressed as mean ± standard deviation (SD). Homogeneity of variances was tested with the Bartlett test at the 0.05 risk error. When homogeneity of variances was respected, One-way ANOVA was performed followed by a Tukey post hoc test. When homogeneity of variances was not respected, which means data did not respect the requirement for ANOVA, Kruskal–Wallis tests were performed at a significance level of 95%. When significant differences appeared, non-parametric multiple comparisons were applied thanks to the Pairwise Multiple Comparison of Mean Ranks Package (PMCMR). The Pearson correlation coefficient (r) was also calculated to observe the relationship between the phenolic content and biological activities (*p* < 0.05).

## 4. Conclusions

Our study demonstrated the possibility of following a sustainable approach in order to valorize two brown macroalgae from Brittany in the cosmetics sector. After an environmentally friendly collection of thalli, without disturbing the life cycle of the alga, we went in search of environmentally friendly extraction and purification processes, together with a follow-up using NMR analysis of extracts/fractions in order to localize phenolic-rich ones. Our study has shown that ^1^H NMR analysis of extracts/fractions is a reliable and inexpensive procedure for detecting those that are rich in phlorotannins, as long as access to an NMR spectrometer is possible. ASE is a fast method, which is automated and does not require large amounts of solvent compared to, for example, the classical maceration. This study demonstrated the interest of ASE in the extraction of active polyphenols from the two species of concern, *Ascophyllum nodosum* and *Halidrys siliquosa*, and more generally from brown seaweeds. For both species, the temperature that permits us to obtain the best phenolic content and activities was 150 °C when compared to 75 °C and 100 °C. On the contrary, the number of ASE cycles had no impact, so one cycle would be enough to extract a high amount of active phenolic compounds. It will be interesting to pursue our investigations in developing a real optimization of conditions applied to the extraction and the purification of phlorotannins in designing our experiment and analyzing our dataset using response surface methodology like in del Pilar Sánchez-Camargo [33] or in Ruiz-Domínguez et al. [75], who carried out this methodology, respectively, in the brown Sargassaceae *Sargassum muticum* and the brown Durvillaeaceae *Durvillaea antarctica,* as examples.

In the aim to find an efficient number of cycles and extraction temperature and in order to follow a sustainable approach (with no impact on the life cycle of species), we used macroalgal samples collected at only one period, which corresponds to no mature thalli in both species. If we had harvested the two species at other times, the thalli would have been mature and we would have found higher levels of phenolic compounds in *A. nodosum* and lower levels in *Halidrys siliquosa*, as reported by Gager et al. [46].

Due to their different biological activities demonstrated in this study, ASE extracts are interesting to valorize in industrial sectors like cosmetics: radical scavenging, reducing and anti-aging activities for both seaweeds and photoprotective activity for *Halidrys siliquosa*. This study also showed an interest in the semi-purification by SPE to enrich fractions in phenolic compounds. Those enriched fractions could be used to further structurally identify phlorotannins contained in *A. nodosum* and *H. siliquosa* with analytical methods such as a combination of HPLC and mass spectrometry.

## Figures and Tables

**Table 1:** ASE extraction mass yield (%) for the two brown macroalgae *Ascophyllum nodosum* (A) and *Halidrys siliquosa* (B) with different cycles (1C = 1 cycle, 1CR = 1 cycle with cell inversion, 2C = 2 cycles, etc.) and temperatures (75 °C, 100 °C and 150 °C). Letters indicate differences between extracts according to temperature for each cycle (One-way ANOVA, *p* < 0.05). No replicate was used for condition 5CR, and then no standard deviation was presented.

Species	Cycle	Temperature (°C)	Yield (%)
*Ascophyllum nodosum*	1C	75	10.9 ± 0.2 *c*
		100	13.2 ± 0.4 *b*
		150	16.5 ± 1.3 *a*
	1CR	75	13.8 ± 0.4 *a*
		100	13.1 ± 4.1 *a*
		150	22.5 ± 1.1 *a*
	2C	75	8.4 ± 3.9 *b*
		100	13.0 ± 1.3 *b*
		150	19.2 ± 0.7 *a*
	2CR	75	20.9 ± 0.6 *b*
		100	22.2 ± 1.1 *b*
		150	26.4 ± 0.3 *a*
	3C	75	15.8 ± 0.7 *c*
		100	19.0 ± 1.1 *b*
		150	24.7 ± 0.3 *a*
	3CR	75	22.0 ± 1.2 *b*
		100	20.6 ± 0.7 *b*
		150	27.6 ± 0.6 *a*
*Halidrys siliquosa*	2C	75	28.4 ± 4.1 *a*
		100	35.6 ± 0.9 *a*
		150	34.0 ± 8.9 *a*
	2CR	75	38.3 ± 0.6 *b*
		100	40.5 ± 0.4 *b*
		150	49.5 ± 2.5 *a*
	3C	75	33.2 ± 0.8 *b*
		100	37.1 ± 1.0 *ab*
		150	38.9 ± 3.2 *a*
	3CR	75	36.5 ± 3.4 *b*
		100	40.3 ± 0.8 *ab*
		150	53.9 ± 9.6 *a*
	5C	75	35.2 ± 1.7 *b*
		100	32.6 ± 0.6 *b*
		150	48.0 ± 0.4 *a*
	5CR	75	38.7
		100	42.5
		150	30.9

**Table 2:** Photoprotective activities as SPF and PF-UVA from *Halidrys siliquosa* ASE extracts obtained with different cycles (1C = 1 cycle, 1CR = 1 cycle with cell turnover, 2C = 2 cycles, etc.) and temperatures (75 °C, 100 °C and 150 °C). Maximal values are written in bold. Letters indicate differences between extracts according to temperature for each cycle (Kruskal–Wallis, *p* < 0.05).

Cycle	Temperature (°C)	SPF	PF-UVA
2C	75	1.32 ± 0.04 *b*	1.12 ± 0.04 *b*
	100	1.29 ± 0.05 *b*	1.17 ± 0.03 *b*
	150	2.33 ± 0.46 *a*	1.85 ± 0.20 *a*
2CR	75	1.30 ± 0.06 *a*	1.19 ± 0.04 *a*
	100	1.22 ± 0.02 *a*	1.12 ± 0.01 *a*
	150	1.57 ± 0.40 *a*	1.27 ± 0.21 *a*
3C	75	1.29 ± 0.04 *b*	1.18 ± 0.03 *b*
	100	1.41 ± 0.06 *b*	1.24 ± 0.04 *b*
	150	1.93 ± 0.49 *a*	1.57 ± 0.40 *a*
3CR	75	1.24 ± 0.03 *b*	1.13 ± 0.02 *b*
	100	1.31 ± 0.03 *b*	1.17 ± 0.02 *b*
	150	2.17 ± 0.15 *a*	1.65 ± 0.13 *a*
5C	75	1.26 ± 0.03 *b*	1.14 ± 0.02 *b*
	100	1.36 ± 0.07 *b*	1.22 ± 0.04 *b*
	150	2.23 ± 0.28 *a*	1.67 ± 0.21 *a*
5CR	75	1.53 ± 0.07 *b*	1.31 ± 0.05 *b*
	100	1.21 ± 0.05 *c*	1.10 ± 0.04 *c*
	150	2.30 ± 0.12 *a*	1.72 ± 0.07 *a*

**Table 3:** Anti-aging activities of 3 extracts of *Ascophyllum nodosum* (An 1C75, 1C100 and 1C150) and *Halidrys siliquosa* (Hs 2C75, 2C100 and 2C150) determined by elastase and tyrosinase inhibitions. Epigallocatechin gallate (EGCG) and kojic acid are positive controls.

Anti-Aging Activities	Extract or Control		Concentrations (mg.mL^−1^)	% of Inhibition
Elastase inhibition	EGCG		1	19.44
	An	1C75	1	57.89
		1C100	1	80.46
		1C150	1	47.39
	Hs	2C75	1	20.60
		2C100	1	14.54
		2C150	1	29.95
Tyrosinase inhibition	Kojic acid		0.1	93.46
	An	1C75	0.1	33.33
		1C100	0.1	12.43
		1C150	0.1	2.48
	Hs	2C75	0.1	15.94
		2C100	0.1	0
		2C150	0.1	42.51

## Data Availability

The dataset generated during the current study is available from the corresponding author upon reasonable request.

## Supplementary Materials

The following supporting information can be downloaded at: https://www.mdpi.com/article/10.3390/md22030112/s1, Table S1: *p*-values obtained with statistical tests (Post hoc after a Kruskal–Wallis test) applied on the SPE fractions for (A) the Total Phenolic Content (TPC), (B) radical scavenging activity by DPPH assay expressed as IC50, and (C) antioxidant activity by FRAP assay expressed as EC50 of *Ascophyllum nodosum* (first number) and *Halidrys siliquosa* (second number), respectively. Significant values (*p* < 0.05) are written in bold. Table S2: Photoprotective activities (SPF and PF-UVA) of one SPE fraction for *Ascophyllum nodosum* (An) (EtOH) and two SPE fractions for *Halidrys siliquosa* (Hs) (Water/EtOH and EtOH) compared to the crude ASE extract. Letters indicate differences between fractions for *H. siliquosa* (Kruskal–Wallis, *p* < 0.005). Figure S1: Entire ^1^H NMR spectra of SPE fractions from *Ascophyllum nodosum* (on the left) and *Halidrys siliquosa* (on the right) compared to the crude extract ASE.
