# Supplementary material for: An Eco-Friendly Extraction and Purification Approach for Obtaining Active Ingredients for Cosmetics from Two Marine Brown Seaweeds
Source: Mar Drugs. 2024 Feb 28;22(3):112. doi: 10.3390/md22030112 (PMC10971643; doi:10.3390/md22030112)
Supplement: Supplementary file 1 [file marinedrugs-22-00112-s001.zip › marinedrugs-2876922-supplementary.pdf]

Supplementary Materials

Table S1. *P*-values obtained with statistical tests (Post-hoc after a Kruskal-Wallis test) applied on the SPE fractions for (A) the Total Phenolic Content (TPC), (B) Radical scavenging activity by DPPH assay expressed as IC50, and (C) antioxidant activity by FRAP assay expressed as EC50 of *Ascophyllum nodosum* (first number) and *Halidrys siliquosa* (second number), respectively. Significant values ($p < 0.05$) are written in bold.

A

TPC	**ASE**	**SPE EtOH**	**SPE EtOH/DCM**	**SPE Water**
**SPE EtOH**	**0.020** / 1.000	X	X	X
**SPE EtOH:DCM**	0.430 / 0.156	**0.003** / 0.441	X	X
**SPE Water**	0.181 / **0.045**	**0.0005** / 0.234	0.996 / 0.996	X
**SPE Water:EtOH**	0.658 / 0.159	0.616 / 0.415	0.102 / **0.006**	**0.032** / **0.001**

B

IC50	**ASE**	**BHA**	**SPE EtOH**	**SPE EtOH/DCM**	**SPE Water/EtOH**	**Vit C**
**BHA**	0.314 / 0.091	X	X	X	X	X
**SPE EtOH**	0.961 / 0.887	0.945 / 0.881	X	X	X	X
**SPE EtOH:DCM**	0.820 / 0.879	**0.036** / **0.020**	0.401 / 0.411	X	X	X
**SPE Water:EtOH**	0.988 / 0.931	0.145 / 0.834	0.744 / 1.000	0.999 / 0.478	X	X
**Vit C**	0.083 / 0.017	0.999 / 0.999	0.709 / 0.618	**0.006** / 0.004	**0.036** / 0.548	X
**Vit E**	0.704 / 0.316	0.999 / 0.995	0.999 / 0.988	0.145 / 0.080	0.401 / 0.970	0.945 / 0.969

C

EC50	**ASE**	**BHA**	**SPE EtOH**	**SPE EtOH/DCM**	**SPE Water/EtOH**	**Vit C**
**BHA**	0.141 / 0.073	X	X	X	X	X
**SPE EtOH**	0.799 / 0.858	0.990 / 0.947	X	X	X	X
**SPE EtOH:DCM**	0.675/ 0.909	**0.020** / **0.031**	0.208 / 0.454	X	X	X
**SPE Water:EtOH**	1.000 / 0.274	0.430 / 1.000	0.927 / 1.000	0.916 / 0.106	X	X
**Vit C**	0.100 / **0.049**	1.000 / 1.000	0.979 / 0.916	**0.014** / **0.022**	0.361 / 1.000	X
**Vit E**	0.429 / 0.541	1.000 / 0.995	1.000 / 1.000	0.073 / 0.224	0.721 / 1.000	0.996 / 0.990

Table S2. Photoprotective activities (SPF and PF-UVA) of one SPE fraction for *Ascophyllum nosum* (An) (EtOH) and two SPE fractions for *Halidrys siliquosa* (Hs) (Water:EtOH and EtOH) compared to the crude ASE extract. Letters indicate differences between fractions for *H. siliquosa* (Kruskal-Wallis, $p < 0.005$).

Seaweeds	Fractions	SPF	PF-UVA
An	ASE 1C150	0	0
	SPE EtOH	1.42 ± 0.04	1.29 ± 0.03
Hs	ASE 2C150	2.33 ± 0.46^{a}	1.85 ± 0.20^{a}
	SPE Water:EtOH	1.17 ± 0.01^{b}	1.10 ± 0.01^{b}
	SPE EtOH	1.82 ± 0.08^{b}	1.47 ± 0.05^{b}

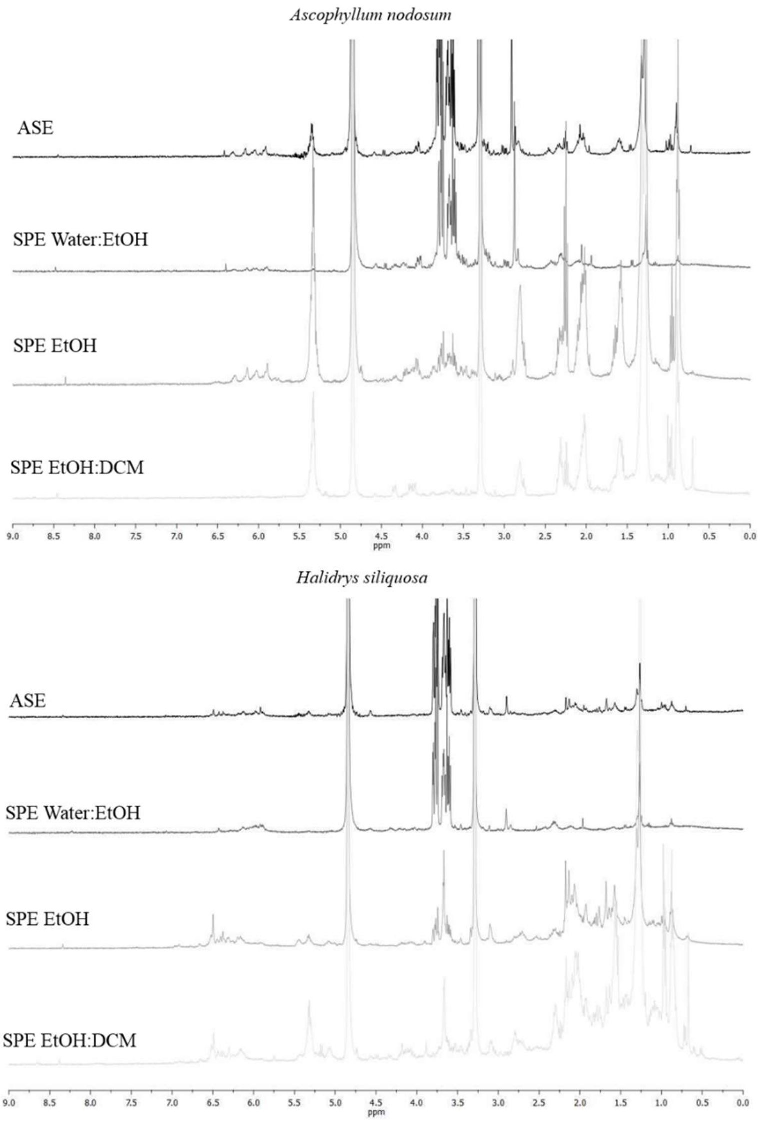

Figure S1: Entire ^{1}H NMR spectra of SPE fractions from *Ascophyllum nodosum* (on the left) and *Halidrys siliquosa* (on the right) compared to the crude extract ASE. ^{1}H NMR spectra were obtained on a Bruker Avance 400 and from samples dissolved within deuterated methanol.
